# Associations between shift work characteristics and work-related accidents and dozing off: combining objective working-time register and retrospective survey data

**DOI:** 10.5271/sjweh.4274

**Published:** 2026-03-01

**Authors:** Bjarte Birkeland Kysnes, Anette Harris, Siri Waage, Erlend Sunde, Ingebjørg Louise Rockwell Djupedal, Ståle Pallesen, Bjørn Bjorvatn, Øystein Vedaa

**Affiliations:** 1 Department of Psychosocial Science, Faculty of Psychology, University of Bergen, Bergen, Norway.; 2 Norwegian Competence Center for Sleep Disorders, Haukeland University Hospital, Bergen, Norway.; 3 Department of Global Public Health and Primary Care, Faculty of Medicine, University of Bergen, Bergen, Norway.; 4 Department of Health Promotion, The Norwegian Institute of Public Health, Bergen, Norway.; 5 Department of Research Administrative Support, Norwegian Institute of Public Health, Norway.; 6 Research Department, Division of Psychiatry, Haukeland University Hospital, Norway.

**Keywords:** adverse event, commute, healthcare worker, injury, late-early, long shift, night shift, safety, shift schedule, short rest period

## Abstract

**Objectives:**

This study investigated the associations between shift work characteristics and self-reported work-related accidents, as well as incidents of dozing off at work and during the work commute.

**Methods:**

Data from a Norwegian hospital’s working-time register encompassed information on quick returns (<11 hours between shifts), day-, evening-, night-, and long (≥12 hours) shifts worked during 2020, and were linked to questionnaire data from 1195 healthcare workers collected in January 2021. The questionnaire assessed work-related accidents causing harm to oneself, patients/others, and/or equipment the last year, as well as dozing off at work the last month and/or during commute the last year. Data were analyzed using negative binomial regressions, adjusting for age, sex, children, marital status, shift work experience, monthly working hours, circadian type, and night shifts.

**Results:**

Number of quick returns the last year was positively associated with causing harm to oneself [incidence rate ratio (IRR) 1.021, 95% confidence interval (CI) 1.009–1.034]. Number of day shifts and evening shifts were negatively associated with causing harm to patients/others (IRR 0.987, 95% CI 0.981–0.992; IRR 0.989, 95% CI 0.982–0.996, respectively). Number of night shifts was positively associated with dozing off at work (IRR 1.005, 95% CI 1.002–1.008) and during commute (IRR 1.007, 95% CI 1.003–1.010), but was not associated with work-related accidents. Number of long shifts was positively associated with healthcare workers causing harm to oneself (IRR 1.198, 95% CI 1.111–1.291), patients/others (IRR 1.209, 95% CI 1.122–1.304), and equipment (IRR 1.174, 95% CI 1.080–1.275).

**Conclusions:**

Limiting quick returns and long shifts may be relevant considerations for improving employee and patient safety.

Shift work refers to work that occurs outside standard hours such as evenings, nights or weekends and is characterized by varying regularity and patterns of shifts (1). In Europe, about 40% of healthcare workers are engaged in shift work (2). Some of the most acute consequences of shift work are reduced sleep duration, disturbed sleep and increased daytime sleepiness (3, 4), as well as fatigue and insufficient rest (5). Consequently, one of the primary concerns with shift work is how it affects employees’ cognitive performance and the risk of work-related accidents (3).

Healthcare workers are vulnerable to several occupational hazards and work-related accidents, including needle stick injury (6), violence (7), and drowsy driving (8), among others. In terms of patient safety, it is estimated that about one in ten inpatients experience adverse events while hospitalized, where events related to surgery, medication/drug administration, and healthcare-associated infections are the most common (9). Risk factors for work-related accidents encompass conditions such as prolonged wakefulness and lack of sleep, monotonous work (time on task), insufficient breaks, and work during the circadian nadir (the time when core body temperature is at its lowest and alertness is reduced), all of which are typically associated with shift work (3).

Quick returns (ie, <11 hours off between two consecutive shifts) is one shift work characteristic that have been associated with curtailed sleep (10, 11). Quick returns are also associated to a higher risk of healthcare workers dozing off at work, causing harm to themselves, patients and equipment (12, 13), as well as experiencing needlestick injuries (14) and other injuries as recorded in national patient and cause of death registries (15, 16).

Night and evening shifts are also associated with elevated risk of work-related accidents or incidents (17, 18). Night work in particular is linked to shortened daytime sleep (3), and night shift workers seem specifically to have an increased risk of dozing off at work (12), drowsy driving (12, 19–21), motor vehicle crashes (19), and causing harm to patients or others (12). Lastly, long working hours (12 hours or longer) have been associated with increased risk of adverse patient outcomes (22), needlestick injuries (14), and drowsy driving (19) among healthcare workers.

Many of the negative aspects of shift work mentioned above vary in terms of their impact on employees’ sleep and health, and are also moderated by individual characteristics (23). Such moderating characteristics include, but are not limited to, age, sex, and circadian type. The latter encompasses low capacity to withstand feelings of lethargy upon losing sleep (languidity), and the ability to work and sleep at odd hours (flexibility) (24).

Few studies have integrated objective data on working hours combined with self-reported work-related accidents and incidents of dozing. Accidents registered in national register-based studies typically capture only severe work-related incidents requiring emergency care or involving fatalities; hence, minor events like needlestick injuries, patient harm, or damage to equipment are typically omitted from such registries. Additionally, previous research, if not register-based, often relies heavily on retrospective self-reports to assess working time exposure (eg 12, 14,).

The present study aimed to expand current knowledge about the relationship between shift work and work-related accidents among healthcare workers, by combining registry data on working hours with self-reported occurrences of work-related accidents and dozing off. We hypothesized that certain shift work characteristics (such as quick returns, evening shifts, night shifts, and long shifts), would be positively associated with self-reported work-related accidents, incidents of dozing off at work and during commutes, while adjusting for age, sex, presence of children in the household, marital status, years of experience with shift work, monthly working hours, circadian type, and night shifts, when relevant.

## Methods

### Design and procedure

This cross-sectional study utilized baseline data from the Health-promoting Work Schedules (HeWoS) randomized controlled trial (25). Objective data on working hours were obtained from local payroll records at Haukeland University Hospital, Bergen, covering the year 2020. These records were then linked to self-reported survey data collected through a digital questionnaire administered in January 2021 via the hospital’s internal IT system, which also served as the platform for recording working hours. All healthcare workers employed in a ≥50.0% position at the hospital units included were invited to participate in the survey (N=2674). In the HeWoS trial, a certain level of homogeneity in the sample was important to reduce possible confounders, thus it was decided to exclude physicians since they have very different scheduling and compensation structures compared to other healthcare workers. A total of 1314 healthcare workers responded to the survey, yielding a response rate of 49.1%. Healthcare workers were included in the analyses if they, on a monthly average, held an employment equivalent to ≥50.0% of a full-time position in 2020, considering both contracted hours and additional/extra shifts. This criterion ensured that healthcare workers had sufficient workload and exposure to shift work. Respondents (N=18) for whom working hour data could not be retrieved were excluded from the analyses. Consequently, the final analytic sample comprised 1195 healthcare workers [85.4% females, mean age 38.3 (SD 12.6) years].

The Regional Committee for Medical and Health Research Ethics in Western Norway approved the HeWoS trial (2020/200386), and the current study only included healthcare workers who provided informed consent.

### Registry data and definition of variables

The registry data on working hours included the actual dates, as well as the start and end times for each shift worked by all healthcare workers. The data were used to calculate the healthcare workers’ average monthly working hours, and the number of quick returns, day shifts, evening shifts, night shifts, and long shifts worked in 2020. The shift work characteristics were classified based on the study by Garde et al (26) with certain modifications. A quick return was defined as having an interval of <11 hours between two consecutive shifts, with each shift lasting ≥3 hours. A day shift was defined as starting after 06:00 and ending before 21:00 hours, with a duration of ≥3 hours. In addition, early morning shifts starting after 03:00 and no later than 06:00 hours were included in the day shift category. An evening shift was defined as involving ≥3 hours of work after 18:00 and before 02:00 hours. A night shift was classified as a work period lasting for ≥3 hours, occurring between 23:00 and 06:00 hours. The long shift was characterized by a shift duration of ≥12 hours but not exceeding 24 hours. Shifts >24 hours were typically related to on-call duty and shifts that allow for sleeping, where the actual workload and rest opportunities of employees are unclear. These shifts were therefore not included in the study. The classified shifts were aggregated to yield a cumulative count of shifts worked by each participant during 2020.

Age was determined from information about birth year obtained from the employee register kept by the hospital, with 2021 serving as the reference year. However, since the data was retrieved in July 2022, healthcare workers who were no longer employed had missing data regarding age (N=32).

### Survey data

The questionnaire assessed demographic characteristics including sex (female/male/do not want to answer), relationship status (living or not living with partner), having children in the household (yes/no), and years of shift work experience. Data for healthcare workers who responded “do not want to answer” regarding sex were set as missing (N=14).

Five items were used to assess the frequency of work-related accidents and incidents of dozing off at work and while driving (27). The questions were phrased as follows: How many times during the last year have you: 1) “experienced work-related accidents that you felt responsible for, causing harm to yourself?” 2) “experienced work-related accidents that you felt responsible for, causing harm to patients/others?” 3) “experienced work-related accidents that you felt responsible for, causing harm to equipment?” 4) “How many times during the last month have you involuntarily dozed off at work?” 5) “How many times during the last year have you dozed off while driving to or from work?” Response options for each item were 0, 1, 2, … 18, 19, 20, 21–30, 31–40, 41–70, 71–100, and >100. An additional composite score indicating overall harm was created for accidents (items 1–3). Due to the characteristics of the response options, the category 21–30 was assigned a recorded value of 21, the 31–40 category received a value of 22, and so forth, resulting in a scale of 0–25, which was utilized in the data analyses.

Circadian type was measured using the revised Circadian Type Inventory, which consists of 11 items assessing the personality trait of flexibility (the ability to work and sleep at odd times), and languidity (the tendency to become tired/sleepy following reduced sleep) (24). Flexibility was assessed by a subscale of five items, with questions such as “Do you enjoy working at unusual times of day or night?” The languidity subscale comprises six items, with “If you go to bed very late do you need to sleep in the following morning?” as an example item. The responses were scored on a 5-point scale ranging from 1 (almost never) to 5 (almost always), with higher scores reflecting a stronger tendency to report the trait. In the current study, the Cronbach’s alpha was 0.76 for the languidity subscale and 0.85 for the flexibility subscale.

### Statistical analyses

The statistical analyses were performed using SPSS Statistics, version 29 (IBM, Armonk, NY, USA). Descriptive statistics were calculated for each variable, including percentages, means and standard deviations (SD). The dependent variables comprised the five types of self-reported frequency of work-related accidents and incidents of dozing off, with mean values close to 0 for all items. For the work-related accident variables, no participants reported values in the higher frequency ranges (eg, 21–30, 31–40 incidents), whereas for the two variables related to dozing-off, six participants (out of a total of 1195) reported values >20. Given the very low frequency of the latter type of responses (0.5% of the sample), any resulting bias is likely to be negligible. Thus, to ensure consistency across analyses, all outcome variables were analyzed as count data. This necessitated the use of a generalized linear model (GLM), such as Poisson or negative binomial regression, to examine the associations between the different shift work characteristics (ie, number of quick returns, day-, evening-, night-, and long shifts over the past year) and work-related accidents and incidents of dozing off. Given the overdispersion in the data (ie, the variance exceeded the mean), the negative binomial regression model was utilized (28), as it provided the best fit in all analyses. Initial crude models were run, followed by adjusted models that controlled for age, sex, presence of children in household, marital relationship status, years of shift work experience, monthly working hours, and circadian type. The inclusion of these confounders was based on previous studies showing that individual characteristics could partially explain the variance in work-related accidents (27, 29, 30). Night shifts were also included as a covariate in all models except when number of night shift was analyzed as the predictor.

Negative binomial regression analyses produce log count estimates as default. In this study, these estimates were converted to incidence rate ratios (IRR), which reflect the multiplicative change in the expected count of the outcome for a one-unit increase in the predictor variable. Hence, the IRR should be interpreted as a relative change in expected counts rather than a change in incidence rate per time unit. Given the high number of statistical tests there is some risk of type I errors (false positives). To address this, multiple testing was adjusted using the Benjamini-Hochberg procedure, applying a false discovery rate (FDR) of 5% (31), and these results were accordingly reported in the present study. Missing data were handled using listwise deletion, resulting in a final sample of N=1118 in the adjusted models.

## Results

Table 1 displays an overview of demographic characteristics and shift work characteristics. During the year, the most frequent shift type were day shifts (mean 82.5, SD 43.6), while long shifts (≥12 hours) were the least prevalent (mean 0.6, SD 1.4). The mean frequency and prevalence of work-related accidents and dozing off while driving to or from work last year, as well as dozing off at work last month, are presented in table 2. The distribution was as expected highly skewed, with mean values close to 0. Still, across the combined items, 40.8% of the healthcare workers reported at least one of the incidents.

**Table 1:** Percentages, means and standard deviation (SD) for demographics and shift work characteristics (N=1195).

Variables		%	Mean (SD)
Sex (N=1181)			
	Female	85.4	
	Male	14.6	
Age (N=1163)			38.3 (12.6)
Marital status			
	Married or living with partner	65.0	
	Living without partner	35.0	
Children in household			
	Yes	34.7	
	No	65.3	
Years of experience (N=1161)			12.0 (10.4)
Monthly hours of work			116.8 (19.9)
Number of shifts worked in 2020			
	Quick returns ^a^		26.4 (16.8)
	Day shifts		82.5 (43.6)
	Evening shifts		49.2 (27.2)
	Night shifts		26.9 (35.2)
	Long shifts (≥12 hours)		0.6 (1.4)

^a^ Quick returns refer to <11 hours of rest between two consecutive shifts.

**Table 2:** Raw means, standard deviation (SD) and prevalences (at least one incident) for self-reported work-related accidents and incidents of dozing off at work and while driving among healthcare workers (N=1195).

	Mean number of times (SD)	Prevalence (%)
Caused harm to yourself last year	0.19 (0.97)	96 (8.0)
Caused harm to patients/others last year	0.18 (0.96)	102 (8.5)
Caused harm to equipment last year	0.15 (0.73)	99 (8.3)
Caused overall harm last year	0.53 (2.21)	196 (16.4)
Dozed off involuntarily at work last month	0.78 (2.14)	294 (24.6)
Dozed off while driving to/from work last year	0.42 (2.06)	132 (11.0)

The results from the crude and adjusted negative binomial regression analyses examining the associations between shift work characteristics and work-related accidents are presented in table 3, while findings related to dozing off are shown in table 4. In the adjusted model after correction for multiple testing, the number of quick returns was positively associated with incidence rate of causing harm to oneself. Conversely, the number of day shifts over the last year was associated with a lower incidence rate of causing harm to patients/others, equipment, and overall harm. Likewise, the number of evening shifts was inversely associated with the incidence rate of causing harm to patients/others. The number of night shifts was unrelated to any of the work-related accident outcomes, but was positively associated with the incidence rate of dozing off both at work and while driving to or from work. The number of long shifts was associated with a higher incidence rate of causing harm to oneself, patients/others, equipment, and overall harm.

**Table 3:** Results from the negative binomial regression analyses examining associations between shift work characteristics and work-related accidents last year among healthcare workers. **Boldface denotes statistical significance** (P<0.05) after controlling for multiple testing, utilizing the Benjamini-Hochberg procedure with a false discovery rate of 5%. [IRR=incidence rate ratios; CI=confidence interval.]

	Caused harm to oneself				Caused harm to patients/others				Caused harm to equipment				Overall harm		
	Crude (N=1195)		Adjusted (N=1118) ^a^		Crude (N=1195)		Adjusted (N=1118) ^a^		Crude (N=1195)		Adjusted (N=1118) ^a^		Crude (N=1195)		Adjusted (N=1118) ^a^
	IRR (95% CI)		IRR (95% CI)		IRR (95% CI)		IRR (95% CI)		IRR (95% CI)		IRR (95% CI)		IRR (95% CI)		IRR (95% CI)
Quick returns ^b^	**1.023 (1.015–1.032)**		**1.021 (1.009–1.034)**		1.003 (0.994–1.012)		0.984 (0.973–0.995)		1.003 (0.993–1.012)		0.987 (0.975–0.999)		**1.011 (1.005–1.017)**		0.998 (0.990–1.006)
Day shifts	1.002 (0.999–1.005)		0.999 (0.994–1.005)		0.997 (0.993–1.000)		**0.987 (0.981–0.992)**		0.997 (0.994–1.001)		**0.988 (0.982–0.994)**		0.999 (0.996–1.001)		**0.992 (0.988–0.995)**
Evening shifts	**1.013 (1.008–1.019)**		1.010 (1.003–1.018)		1.002 (0.966–1.007)		**0.989 (0.982–0.996)**		1.004 (0.998–1.010)		0.995 (0.988–1.003)		**1.007 (1.003–1.011)**		0.998 (0.993–1.003)
Night shifts	0.994 (0.990–0.999)		0.996 (0.991–1.002)		0.997 (0.992–1.001)		0.999 (0.993–1.005)		0.995 (0.990–1.000)		0.996 (0.990–1.003)		0.995 (0.992–0.999)		0.997 (0.993–1.000)
Long shifts (≥12 hour)	**1.220 (1.137–1.310)**		**1.198 (1.111–1.291)**		**1.187 (1.109–1.271)**		**1.209 (1.122–1.304)**		**1.192 (1.105–1.287)**		**1.174 (1.080–1.275)**		**1.207 (1.142–1.275)**		**1.186 (1.117–1.259)**

**Table 4:** Results from the negative binomial regression analyses examining associations between shift work characteristics and incidents of dozing off in the last month/year among healthcare workers. Boldface denotes statistical significance (P<0.05) after controlling for multiple testing, utilizing the Benjamini-Hochberg procedure with a false discovery rate of 5%. [IRR=incidence rate ratios; CI=confidence interval.]

	N Crude/Adjusted	Dozed off involuntarily at work last month				Dozed off while driving to/from work last year		
		Crude		Adjusted ^a^		Crude		Adjusted ^a^
		IRR (95% CI)		IRR (95% CI)		IRR (95% CI)		IRR (95% CI)
Quick returns ^b^	1195/1118	0.998 (0.993–1.003)		1.003 (0.995–1.010)		**0.986 (0.979–0.992)**		0.995 (0.985–1.005)
Day shifts	1195/1118	0.997 (0.995–0.999)		1.000 (0.996–1.004)		0.997 (0.995–1.000)		0.998 (0.993–1.003)
Evening shifts	1195/1118	0.998 (0.995–1.001)		1.000 (0.996–1.005)		**0.989 (0.985–0.993)**		0.993 (0.987–0.999)
Night shifts	1195/1118	1.003 (1.001–1.005)		**1.005 (1.002–1.008)**		1.001 (0.999–1.004)		**1.007 (1.003–1.010)**
Long shifts (≥12 hours)	1195/1118	1.026 (0.967–1.088)		1.067 (1.001–1.137)		0.994 (0.924–1.070)		1.072 (0.982–1.158)

^a^ Adjusted for age, sex, children in household, marital status, year of experience, monthly hours of work, night shifts and circadian type. Night shifts excluded as covariate when analyzed as predictor. ^b^ Quick returns refer to <11 hours of rest between two consecutive shifts.

In the adjusted model, each one-unit increase in quick returns was associated with an expected log count increase of 0.021 for causing harm to oneself (IRR 1.021). The model estimates were used to calculate the expected IRR for an average number of quick returns. The calculations showed that the average of 26.4 quick returns last year was associated with 74.0% more incidents of causing harm to oneself, compared to working no quick returns. Conversely, compared to no day shifts, the annual average of 82.5 day shifts was associated with 66.0%, 63.0%, and 48.0% fewer incidents of causing harm to patients/others, equipment, and overall harm, respectively. The average of 49.2 evening shifts during a year was associated with 42.0% fewer incidents of causing harm to patients/others, compared to no evening shifts. Further, the average of 26.9 night shifts in 2020 was associated with 21.0% more incidents of dozing off while driving to or from work and 14.0% more incidents of dozing off at work, relative to no night shifts. As participants worked an average of 0.6% long shifts, the effect of one long shift was considered more interpretable than calculating the average. One long shift was associated with higher incidents of causing harm to oneself (22.0%), patients/others (23.0%), equipment (19.0%), and overall harm (20.0%), compared to no long shifts.

## Discussion

### Main findings

The purpose of this study was to generate knowledge about the relationship between shift work characteristics and self-reported work-related accidents, dozing off at work, and while driving to or from work, among healthcare workers. After adjusting for covariates and multiple testing, the results indicated that the number of quick returns was associated with a higher incidence rate of causing harm to oneself, as hypothesized. Conversely, inverse associations were found between the number of day shifts and incidents of causing harm to patients/others, equipment, and overall harm, as well as between the number of evening shifts and incidents of causing harm to patients/others. Number of night shifts was associated with a higher incidence rate of dozing off at work/while driving but not associated with work-related accidents. The frequency of long shifts was associated with a higher incidence rate of causing harm to oneself, patients/others, equipment, and overall harm.

### Comparison with previous studies and interpretations

The results showed that quick returns were linked to a higher incidence rate of healthcare workers causing harm to themselves, which is in line with previous studies based on either questionnaire data (12–14) or national registers (16). On average, employees in the current study had 26.4 quick returns during the exposure year, which corresponded to a 74.0% increase in incidents of harming oneself compared to the incidence rate that is already there for employees with no quick returns. Although this is an observational study, precluding conclusions about causality, it suggests that quick returns represent a significant risk factor for employee safety, adding to the substantial financial and health-related burden of occupational accidents (32). In terms of possible mechanisms, short sleep duration and increased sleepiness/fatigue are likely candidates (10, 33, 34). Interestingly, quick returns were not associated with a higher incidence of healthcare workers causing harm to patients/others or causing harm to equipment. This finding was somewhat surprising, given that previous studies have relatively consistently demonstrated a higher risk of such adverse events when exposed to quick returns (12, 13). One possible explanation for the discrepancy between previous and the current study is methodological differences, as prior studies using the same questionnaire-based measures typically have relied on retrospective self-reports of the number of quick returns worked over the last year. In contrast, the present study employed objective registry records to determine the number of quick returns, thus providing an accurate assessment of shift work exposure. It is also conceivable that employees find it easier to self-report accidents involving themselves rather than those involving others or equipment, as the latter may be considered more sensitive or even taboo. This may be reflected in a fear of negative consequences that has been noted as a barrier to incident reporting among nurses (35).

Both the number of day and evening shifts were associated with fewer incidents of causing harm to patients/others. Additionally, a higher number of day shifts was associated with fewer incidents of causing harm to equipment. However, evening shifts were not positively associated with either work-related accidents or dozing off, contradicting previous robust studies (16, 36). The outcome measures of the previous studies only include work-related accidents leading to a hospital visit. Thus, potential minor workplace incidents reported by participants in the current study might provide another insight. Indeed, as recently noted in a systematic review (18), previous research shows more mixed results regarding the link between evening shifts and accidents, compared to night shifts and accidents. Considering that the circadian rhythm promotes alertness during daytime and early evening hours (37), one might overall expect lower incidence rates during daytime. Further, day and evening shifts, by their nature, do normally not lead to sleep deprivation. In addition, these shifts are typically characterized by higher staffing levels and greater support structures compared to night shifts. Day shifts, although busier (38), can thus benefit from more frequent communication, collaboration and coworker monitoring/surveillance, likely preventing accidents and facilitating more rapid responses in cases of emerging issues.

Conversely, evening shifts may involve fewer scheduled activities, but more time spent on occasional procedures compared to day shifts (38). One might argue that a potential lighter workload, combined with relatively high circadian alertness, may partly account for the absence of an association between evening shifts and increased accident proneness observed in the current study. Nonetheless, day shifts often involve a higher frequency of patient interactions and procedures (38), which increases the base rate for potential errors. Despite these considerations, the current findings suggest that day and evening shifts are not associated with a higher risk of accidents; rather, they appear to be associated with a lower risk.

Number of night shifts was associated with a higher risk of dozing off while driving to or from work, consistent with previous diary studies (19, 20) as well as a study using objective measures of drowsiness among nurses (21). The current study extends these findings by demonstrating similar results using objective registry data together with self-reported questionnaire data. Possible mechanisms involved may include extended wakefulness and staying awake during the circadian trough of alertness (typically around 04:00–05:00 hours), which is particularly pertinent during the night shift (3, 4). The present results underscore the need for heightened attention to healthcare workers with frequent night shifts to mitigate potential accident risks for both employees and the public (19, 20).

The frequency of night shifts was also associated with a higher incidence rate of involuntarily dozing off at work. While dozing off at work can pose risks for negligence or workplace accidents, it can also serve a rejuvenating function that in turn can enhance alertness and performance, and as such, potentially reducing the risk of fatigue and accidents (39). Still, involuntarily dozing off at work clearly may pose a greater risk of adverse events, as opposed to planned or scheduled napping, which may be beneficial for alertness and performance (40). Given the phrasing of the questionnaire, it is unclear how the healthcare workers interpreted it, including whether they considered incidents of voluntary or involuntary dozing off when responding.

Although prior research often links night work to more accidents (eg 18, 41,), we found no association in the present study (beyond dozing). This may reflect a healthy-worker effect, where frequent night workers are those who cope well, making harms harder to detect. Yet this contrasts with our separate finding of higher incident rate of dozing-off among night shift workers. The basis for this inconsistency is not evident.

The higher incidence rates associated with number of long shifts are in line with the literature, noting that long shifts lasting ≥12 hours might elevate the risk of work-related accidents (14, 17, 22, 41, 42). Relevant mechanisms comprise extended wakefulness, which may cause insufficient rest and fatigue (5). It is important to note that long shifts may have occurred more frequently in 2020 compared to before, due to COVID-19, when healthcare workers were assigned to extraordinary duty. The long shifts may in several cases represent overtime work that was unplanned, which may be relevant for the interpretation of the current findings.

### Strengths and limitations

A notable strength of the current study was the use of objective working time data to assess exposure to different shifts worked during the last year, complemented by questionnaire data on both work-related accidents and episodes of dozing off. Furthermore, the study accounted for circadian type, which recently was found to be related to shift workers’ alertness and accident risk (30). Another asset of the study was the high response rate (49.1%), which is higher than what many contemporary surveys achieve (43). Nonetheless, even with this comparatively robust participation, some uncertainty remains regarding selection and the generalizability of the findings, and caution is thus warranted when interpreting the results. The retrospective self-reported outcome measures of accidents/dozing are prone to individual subjectivity and recall bias. While the questions regarding work-related accidents and dozing off have been utilized in previous studies (12, 27), it is important to note that these items have not undergone formal validation. A possible limitation of this study is that the different shifts studied are inevitably related to each other (eg, night shifts are often followed by days off, and day shift may be followed by evening shifts), which is not fully accounted for in the analyses (except that we adjust for night shifts). We believe that further adjustment (beyond night shifts) would lead to over-adjustment in the analyses, since several of the key shift work characteristics, such as quick returns and long shifts, are composed of individual day, evening and night shifts. Furthermore, the cross-sectional design of the current study limited the ability to draw conclusions about causal relationships and does not allow to rule out reverse causality.

Another limitation to consider concerns the variation in workload across different shifts. It has been observed that inpatients typically require less intensive care and fewer procedures during night shifts compared to day and evening shifts (38). In the current study, we were unable to adjust for such workload variations. This could be a significant confounder, as a systematic review on workload in intensive care units suggests that increased nursing workload negatively impacts patient outcomes (44). Future studies should thus aim to measure and adjust for workload/number of procedures performed as well as other relevant occupational and operational confounders. Furthermore, linking objective records of working hours and hospitals’ incident reporting systems (adverse events affecting employees, patients, or equipment) in future studies could provide more precise and richer data. This could ensure a closer link between the time of exposure to certain shift types and schedules, and the time point of reported incidents.

### Concluding remarks

Taken together, our findings suggest that work schedules that restrict time for recovery – in particular quick returns, long shifts, and night work-related sleepiness – are more strongly associated with self-reported work-related accidents than the overall frequency of day or evening shifts. This pattern indicates that promoting sufficient rest opportunities and limiting quick returns and very long shifts may be important strategies for improving both employee safety and patient safety in the healthcare sector. Future studies should measure and adjust for variation in workload and examine whether similar associations are observed in other occupational sectors. There is also a need for more precise data capturing minor workplace incidents that are currently missed by national registers, which typically only record severe cases requiring emergency care or involving fatalities. Implementing effective hospital-based incident-reporting systems, supported by a strong reporting culture, is therefore important to improve data validity and reduce reliance on retrospective self-reports, as used in the present study. Given the observational design and remaining uncertainty about selection and measurement error, the findings should be interpreted as indicative of associations rather than causal effects.
